# Fluorescent Biosensors for Neurotransmission and Neuromodulation: Engineering and Applications

**DOI:** 10.3389/fncel.2019.00474

**Published:** 2019-10-23

**Authors:** Anna V. Leopold, Daria M. Shcherbakova, Vladislav V. Verkhusha

**Affiliations:** ^1^Medicum, Faculty of Medicine, University of Helsinki, Helsinki, Finland; ^2^Department of Anatomy and Structural Biology, Gruss-Lipper Biophotonics Center, Albert Einstein College of Medicine, Bronx, NY, United States

**Keywords:** GPCR, GltI, GABA, glutamate, dopamine, serotonin, norepinephrine, neural circuit

## Abstract

Understanding how neuronal activity patterns in the brain correlate with complex behavior is one of the primary goals of modern neuroscience. Chemical transmission is the major way of communication between neurons, however, traditional methods of detection of neurotransmitter and neuromodulator transients in mammalian brain lack spatiotemporal precision. Modern fluorescent biosensors for neurotransmitters and neuromodulators allow monitoring chemical transmission *in vivo* with millisecond precision and single cell resolution. Changes in the fluorescent biosensor brightness occur upon neurotransmitter binding and can be detected using fiber photometry, stationary microscopy and miniaturized head-mounted microscopes. Biosensors can be expressed in the animal brain using adeno-associated viral vectors, and their cell-specific expression can be achieved with Cre-recombinase expressing animals. Although initially fluorescent biosensors for chemical transmission were represented by glutamate biosensors, nowadays biosensors for GABA, acetylcholine, glycine, norepinephrine, and dopamine are available as well. In this review, we overview functioning principles of existing intensiometric and ratiometric biosensors and provide brief insight into the variety of neurotransmitter-binding proteins from bacteria, plants, and eukaryotes including G-protein coupled receptors, which may serve as neurotransmitter-binding scaffolds. We next describe a workflow for development of neurotransmitter and neuromodulator biosensors. We then discuss advanced setups for functional imaging of neurotransmitter transients in the brain of awake freely moving animals. We conclude by providing application examples of biosensors for the studies of complex behavior with the single-neuron precision.

## Introduction

**FIGURE 1:**
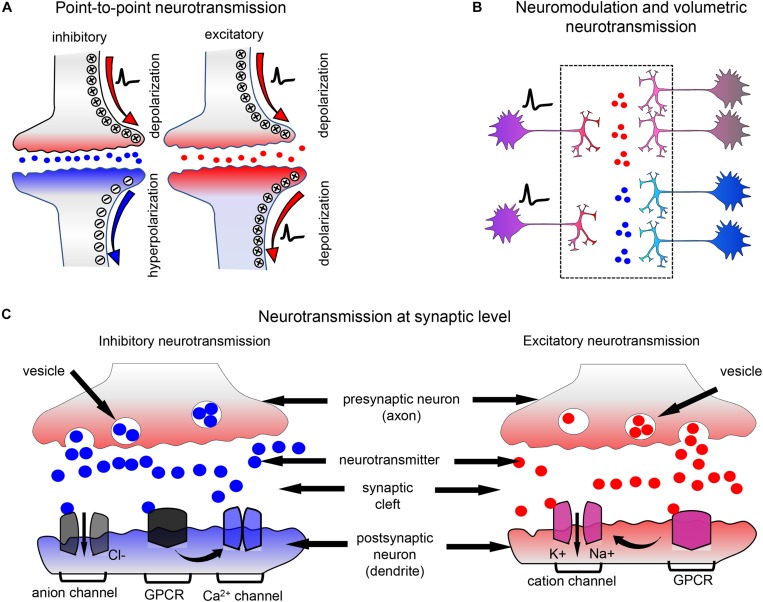
Neurotransmission and neuromodulation principles. **(A)** In local neurotransmission, neurotransmitters packed in vesicles are released into the synaptic cleft and interact with the ionotropic receptors of neurotransmitters, which are typically ion channels. The interaction causes receptor with inhibitory or excitatory neurotransmitter channel to open to negatively or positively charged ions. The post-synaptic neuron is inhibited (blue) or excited (red). Neurotransmitters released by a presynaptic neuron act only on the single post-synaptic neuron and, after interaction with ionotropic receptors, are rapidly destroyed in the synaptic cleft. **(B)** In neuromodulation and volumetric transmission, neuromodulators released by a single neuron act simultaneously on the groups of neurons, modulating their synaptic strength. **(C)** Neurotransmission at synaptic level: on the left: the inhibitory neurotransmitter is released in the synaptic cleft and activates anion channels and GPCR receptors. Activation of GPCR receptors by the inhibitory neurotransmitters, such as GABA, negatively regulates calcium channels. On the right: the excitatory neurotransmitter is released in the synaptic cleft and activates cation channels and relevant GPCR receptors.

Neurotransmitters and neuromodulators are chemicals, which are crucial for signal transmission in neuronal circuits. Neurotransmitters are released by the axon of the presynaptic neuron and excite, like glutamate, or inhibit, like γ-aminobutyric acid (GABA), the adjacent neurons in a sub-second timescale. Neurotransmitters are stored in vesicles in presynaptic terminals and are released into the synaptic cleft in response to an action potential (Figure 1A; Klein et al., 2019). Neuromodulators are diffusing chemicals that modulate activity of the groups of neurons and can act not only on fast but also on slow timescales (Figures 1B,C). However, even classical fast neurotransmitters, such as glutamate, may not necessarily act as point-to-point transmitters; and diffusion of neurotransmitters from the synaptic cleft to the extracellular space is sufficient to activate non-synaptic receptors at a significant distance. This type of neurotransmission is called volumetric transmission (Taber and Hurley, 2014).

Neurotransmitters act on ionotropic and metabotropic receptors. Ionotropic receptors are ion channels whose activity is directly modulated by neurotransmitters. As an example, glutamate interaction with its ionotropic receptors (iGluRs) at the plasma membrane of the post-synaptic neurone leads to opening of channel pores, cation influx and membrane depolarization (Figure 1C; Traynelis et al., 2010). Metabotropic receptors are G-protein-coupled receptors (GPCRs) and their activation by neurotransmitters leads to indirect modulation of ion channels activity via activation of G-protein signaling (Nadim and Bucher, 2014).

Neuromodulators act mostly through G-protein-coupled receptors (Figure 1C; Nadim and Bucher, 2014; Avery and Krichmar, 2017), however, clear distinction between neurotransmitters and neuromodulators is problematic, as far as many classical neuromodulators may act through ionotropic receptors. For example, acetylcholine acts mostly as point-to-point neurotransmitter at neuromuscular junctions and in peripheral nervous system but as neuromodulator in central nervous system (Picciotto et al., 2012).

While neurotransmission leads to the fast excitation or inhibition of the post-synaptic neurons, neuromodulation results in the alteration of synaptic efficacy and in the changes of synaptic dynamics. Action of neuromodulators can change the rates of depression and facilitation at synapses, allowing synaptic dynamics as well as strength to vary (Nadim and Bucher, 2014). In the nervous system neuromodulators regulate switching of brain states, with the examples of serotonin controlling mood and norepinephrine controlling sleep and arousal (Avery and Krichmar, 2017).

All neurotransmitters and neuromodulators are essential to cognition and behavior (Nadim and Bucher, 2014; Avery and Krichmar, 2017). The correlation of chemical transmission in animal brain with its complex behavior can be studied using modern fluorescent biosensors. These biosensors provide high spatiotemporal precision for the visualization of fast neurotransmitter transients in neural circuits in brains of behaving animals (Brunert et al., 2016; Xie et al., 2016; McGirr et al., 2017; Sun et al., 2018; Tanaka et al., 2018; Feng et al., 2019; Marvin et al., 2019).

Fluorescent proteins (FPs) are essential part of modern biosensors. There are two major approaches of using FPs in biosensors. The first approach employs Forster resonance energy transfer (FRET) between two FPs (Li et al., 2016). FRET occurs when a donor FP is excited by light and non-radiatively transfers the excitation energy to the nearby chromophore, an acceptor. The second approach employs circular permutants of FPs (cpFPs) (Wang et al., 2018). Circular permutation involves rearrangement of the parts of the original FP that retains the protein secondary structure. Certain regions in cpFPs tolerate insertion of other proteins; and conformational changes in the insert profoundly influence the fluorescence intensity. Moreover, circular permutants alter the relative orientation of the chromophore to a fusion partner, which is exploited in the optimization of FRET-based biosensors by inserting cpFPs.

Fluorescent biosensors can be delivered to the animal brain using viral vectors and detected in behaving animals by fiber photometry, including multi-channel fiber photometry (Guo et al., 2015), stationary two-photon (2P) excitation microscopy (Svoboda and Yasuda, 2006), and miniaturized head-mounted microscopes (Aharoni et al., 2019). Imaging of neurotransmitter transients in response to visual, audio or olfactory stimuli can be performed in restrained animals, however, head-mounted wireless miniaturized microscopes allow imaging of biosensors in the brain of freely moving animals (Liberti et al., 2017).

In this review, we firstly summarize available fluorescent biosensors for neurotransmitters and neuromodulators. We then outline a biosensor engineering workflow and provide the basic design principles for the modern biosensors. Next, we overview detection and functional imaging techniques that allow recording neurotransmitter and neuromodulator transients in animals. We then discuss how the biosensors enable monitoring brain function with high spatiotemporal precision and how they can be combined with common optogenetic tools for all-optical electrophysiology assays. Lastly, we outline avenues for engineering and applications of future biosensors for neurotransmitters and neuromodulators.

For biosensors of neural activity, such as genetically encoded membrane voltage and calcium indicators, we refer our readers to the recent reviews (Chen et al., 2017; Bando et al., 2019; Kannan et al., 2019; Piatkevich et al., 2019).

## General Designs of Biosensors for Chemical Transmission

To develop fluorescent biosensor for neurotransmitter or neuromodulator, a FRET pair of FPs or a cpFP is combined with a respective binding protein, called a sensing domain. In FRET-based biosensors a ratio between fluorescence intensities of the FRET donor and FRET signal changes upon neurotransmitter binding, therefore they are referred as ratiometric biosensors. In single-FP-based biosensors, fluorescence at single wavelength changes upon neurotransmitter binding, therefore they are referred as intensiometric biosensors (Lindenburg and Merkx, 2014; Chen et al., 2017). Currently, two types of sensing domains are used for engineering of both ratiometric and intensiometric biosensors.

**FIGURE 2:**
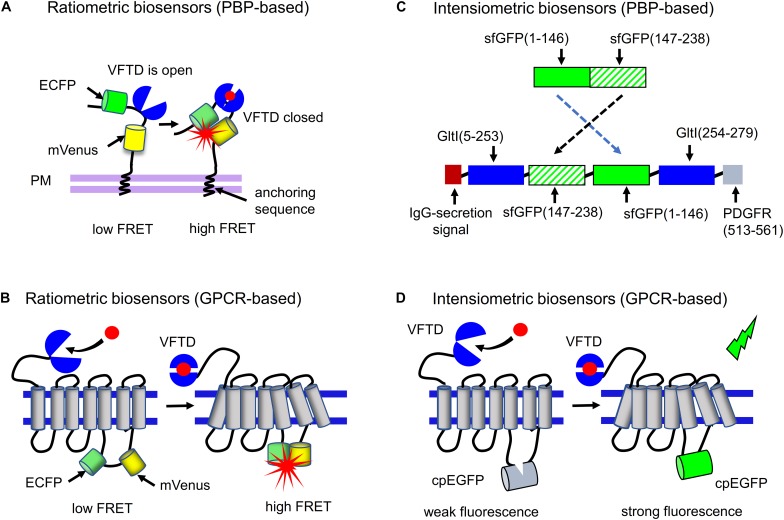
Ratiometric and intensiometric biosensors. **(A)** FRET-based biosensors in which PBP proteins are used as neurotransmitter-sensing domains. A PBP is inserted between FRET pair of FPs, such as ECFP and mVenus. Upon PBP interaction with neurotransmitter the FRET efficiency between FPs changes, which can be detected. **(B)** FRET-based biosensors in which GPCR receptors are used as neurotransmitter-sensing domains. A FRET pair consisting of ECFP and mVenus is inserted in the third intracellular loop of the respective GPCR. Upon interaction of the receptor with neurotransmitter FRET between two FPs changes. **(C)** Example of the PBP-based intensiometric biosensor is shown. Circular permutant of superfolder GFP (sfGFP) between positions 146–147 is inserted into GltI protein, resulting in the SF-iGluSnFR glutamate biosensor (Marvin et al., 2018). N-terminal IgG-secretion signal ensures transport of the biosensor to extracellular space, while C-terminal transmembrane domain of PDGFR receptor anchors the biosensor to the plasma membrane **(D)** Example of the GPCR-A-based intensiometric biosensor is shown. Circularly permuted EGFP (cpEGFP) is inserted into the third intracellular loop of GPCR having the VFTD domain at the N-terminus. After binding neurotransmitter or neuromodulator, the conformation of GPCR changes that results in enhancement of cpEGFP fluorescence.

First, periplasmic-binding proteins (PBPs) that interact with neurotransmitters are used. PBPs possess so-called Venus Flytrap Domain (VFTD), which changes its conformation upon binding neurotransmitter. VFTD is a bilobal protein, which remains “open” in the inactive state and “closes” upon ligand binding (Figure 2; Kunishima et al., 2000; Pin et al., 2003). At the moment only three PBP proteins, such as GltI from *Escherichia coli*, Atu2422 from *Agrobacterium tumefaciens*, and Pf622 from *Pseudomonas fluorescens* are used in the neurotransmitter biosensors (Table 1). In PBP-based biosensors VFTD domain is inserted into the FRET pair (Figure 2A; Chen et al., 2017) or, on the contrary, cpFP is inserted into the flexible region of VFTD domain (Figure 2C; Marvin et al., 2013). Upon neurotransmitter binding, VFTD domain changes its structure from “open” to “closed” and that results in the either FRET between fluorescent proteins or restoration of the cpFP fluorescence, if cpFP is used.

**TABLE 1:** Modern biosensors for neurotransmitters and neuromodulators.

**FRET-based biosensors**	**FRET pair**	**Relative change of FRET ratio,**ΔR/R **(%)**	**K_d_ (μM)**	**Conditions used to measure ΔR/R and K_d_**	**Substance (neurotransmitter or neuromodulator)**	**Template for sensing domain**	***In vivo* use**	**References**
SuperGluSnFR	ECFP-Citrine	44	2.5	Cultured neurons, 1P microscopy	Glutamate	GltI	Not tested	Hires et al., 2008
M1-cam5	ECFP-EYFP	10	Not determined	HEK293 cells, 1P microscopy	Acetylcholine	M1mAChR		Markovic et al., 2012
GlyFS	EGFP-Venus	20	20	Brain slices, 2P microscopy	Glycine	Atu2422 (AYW mutant)		Zhang et al., 2018

**Single-FP-based biosensors**	**Circularly permuted FP**	**Relative change of fluorescence,**ΔF/F **(%)**	**K_d_ (μM)**	**Conditions used to measure ΔF/F and K_d_**	**Substance (neurotransmitter or neuromodulator)**	**Template for sensing domain**	***In vivo* use**	**References**

iGluSnFR	cpGFP	103	4.9	Cultured neurons, 1P microscopy	Glutamate	GltI	Imaging of dendritic spines	Marvin et al., 2013
SF-iGluSnFR A184V	sfGFP	69	0.6					Marvin et al., 2018
SF-iGluSnFR S72A		250	34					
SF-Azurite-iGluSnFR	Azurite	66	46					
SF-Venus-iGluSnFR	Venus	66	2					
SF-mTurquoise2-iGluSnFR	mTurquoise	90	41					
iGABASnFR	sfGFP	250	9	Purified protein, fluorimeter	GABA	Pf622	Imaging of single neurons	Marvin et al., 2019
iGlu_f_	EGFP	100	137	HEK293 cells, stopped-flow	Glutamate	GltI	Not tested	Helassa et al., 2018
iGlu_u_		170	600					
R-iGluSnFR1	mApple	−33	11	Purified protein, fluorimeter				Wu et al., 2018
R-ncp-iGluSnFR1			0.9					
GACh	EGFP	90	2	HEK293 cells, 1P microscopy	Acetylcholine	M_3_R	Imaging of single neurons	Jing et al., 2018
GRAB_NE__1__m_		230	1.9		Norepinephrine	α2AR	Aggregated fluorescence signal	Feng et al., 2019
GRAB_NE__1__h_		150	0.093					
Nb80-GFP		Not determined	Not determined	Not applicable		β2AR/Nb80	Not tested	Irannejad et al., 2013
OR-sensor	EGFP	Not determined	Not determined	Not applicable	Activation of μ and δ ORs	μ and δ ORs/Nb33	Not tested	Stoeber et al., 2018
iATPSnFR	spGFP	150	630	Cultured neurons, 1P microscopy	ATP	ε subunit of FOF1 ATPase from *Bacillus* PS3	Imaging of single astrocytes	Lobas et al., 2018
dLight1.1	EGFP	230	0.33	HEK293 cells, 1P microscopy	Dopamine	DRD1 (inserted into the ICL3)	Aggregated fluorescence signal	Patriarchi et al., 2018
dLight1.2		340	0.77					
DA1m		90	0.13			DRD2 (inserted into the ICL3)		Sun et al., 2018
DA1h			0.01					
								

Glutamate, acetylcholine, serotonin and GABA binding-proteins were discovered in organisms lacking nervous systems and even in unicellular organisms (Venter et al., 1988; Hoyle, 2011; Elphick et al., 2018). Bacteria use neurotransmitter-recognizing motifs presented by PBPs (Moussatova et al., 2008) in ATP binding cassette (ABC) transporters (Wilkens, 2015), with an example of several glutamine, L-histidine, glycine, and arginine-binding transporters (Moussatova et al., 2008). Moreover, the family of plant glutamate-receptor like proteins might become a source of neurotransmitter-binding motifs. Proteins of this family bind GABA and various amino acids, such as glycine and glutamate (Forde and Roberts, 2014). AtGAT1, a high-affinity GABA transporter in *Arabidopsis thaliana* may also present a valuable scaffold for engineering of GABA biosensors (Meyer et al., 2006). Dopamine/norepinephrine transporter (SmDAT) (Larsen et al., 2011), as well as transporters of serotonin and norepinephrine (Ribeiro and Patocka, 2013), were found in human parasite trematode *Schistosoma mansoni*.

Second, GPCRs are used for the development of neurotransmitter and neuromodulator biosensors. GPCRs are membrane-spanning proteins, which change their conformation upon binding of neurotransmitters or neuromodulators and activate downstream signaling (Niswender and Conn, 2010). In this type of biosensors the activation state of GPCR is detected. Neurotransmitters and neuromodulators mostly interact with GPCRs of the A group (GPCR-A), with the exception of glutamate and GABA, which interact with the GPCRs of the C group (GPCR-C), such as metabotropic glutamate receptors and GABA receptors. GPCR-C receptors possess the large N-terminal domain, which is structurally similar to the VFTD of PBPs. Similar to VFTDs of GltI and Atu2422, the VFTD of GPCR-C remains “open” in the inactive state and “closes” upon neurotransmitter binding (Figure 2B). Second and third intracellular loops of GPCR-C receptors form the cavity, responsible for the G-protein recognition (Pin et al., 2003). The GPCR-A group comprises all other receptors of neurotransmitters and neuromodulators, including catecholamine aminergic receptors, β-adrenergic receptors, histamine H1 receptor and muscarinic acetylcholine receptors (Katritch et al., 2013). As opposed to GPCR-C receptors, GPCR-A receptors have the longer intracellular third domain (it is the longest intracellular domain in these receptors, while in GPCR-Cs the second domain is the longest) and do not have VFTD domain.

In both GPCR-A- and GPCR-C-based neurotransmitter biosensors, a FRET pair of FPs (Figure 2B) or a cpFP (Figure 2D) is inserted in a third intracellular loop of the receptor (Sun et al., 2018; Wang et al., 2018; Feng et al., 2019). Upon neurotransmitter binding to the extracellular part of the GPCR, the receptor conformational changes are transferred to the intracellular part, causing the FRET changes between FPs or the recovery of cpFP fluorescence.

Below we describe the modern biosensors for neurotransmitters and neuromodulators, which are compatible with the studies of chemical transients in the brain of behaving animals.

## Biosensors for Glutamate

Glutamate is the most abundant excitatory neurotransmitter in the mammalian nervous system. Concentration of glutamate is tightly regulated by the number of transporters and glutamate-degrading enzymes, preventing the glutamate excitotoxicity (Zhou and Danbolt, 2014). Glutamate is released not only by neurons but also by glial cells (Harada et al., 2015). As it has been mentioned, glutamate-binding proteins are found in all kingdoms of life (Pin et al., 2003; Ribeiro and Patocka, 2013; Forde and Roberts, 2014). Some of these proteins, such as GltI, are similar to extracellular VFTD domains of mammalian GPCR-C receptors of glutamate and GABA (Figure 2A).

The first glutamate ratiometric biosensor was based on the GltI inserted between the ECFP-mVenus FRET pair of proteins (Okumoto et al., 2005). It was later improved by replacing mVenus with EYFP and systematic screening for the highest glutamate sensitivity. This resulted in SuperGluSnFR biosensor (Table 1) that exhibits 44% change in FRET/donor ratio upon glutamate binding (Hires et al., 2008).

Later, an intensiometric glutamate sensor, iGluSnFR, was engineered by inserting of cpEGFP in GltI (Figure 2C; Marvin et al., 2013). Although iGluSnFR was shown to work *in vivo*, it has some limitations including slow readout of glutamate dynamics in synapses and inability to detect sparse glutamate release (Marvin et al., 2018). iGluSnFR was recently improved by developing two fast glutamate biosensors iGlu_u_ and iGlu_f_ (Helassa et al., 2018). Reducing the GltI affinity to glutamate enabled increasing rate of its dissociation in iGlu_u_ and iGlu_f_. Another family of glutamate biosensors was developed by replacing cpEGFP in iGluSnFR with circularly permuted superfolder GFP (fGFP) (Marvin et al., 2013). Moreover, iGluSnFR affinity to glutamate was changed by mutating GltI in the ligand-binding center. This resulted in two biosensors, SF-iGluSnFRS72A and SF-iGluSnFRA184V, with the reduced and enhanced affinity to glutamate, respectively. To develop multicolor SF-iGluSnFR biosensors, Marvin et al. (2018) introduced in the cpsfGFP chromophore mutations from spectrally-shifted GFP variants, such as mAzurite, mTurquoise2 and mVenus, resulting in the blue, cyan and yellow SF-GluSnFRA184V variants, respectively.

To shift intensiometric glutamate biosensors toward red spectral range, cpEGFP in iGluSnFR was replaced with circularly permuted mApple red FP, resulting in R-iGluSnFR1 and R-ncp-iGluSnFR1 biosensors (Wu et al., 2018). Although R-iGluSnFR1 exhibits the high glutamate affinity and dynamic range (Table 1; Wu et al., 2018), it demonstrates the fluorescence decrease upon glutamate binding, as opposed to the glutamate biosensors based on the GFP variants. It makes R-iGluSnFR1 rather similar to “turn-off” quantum dots used in neurotransmitter research, which lose fluorescence upon neurotransmitter binding (Ankireddy and Kim, 2015). Red fluorescent biosensors for glutamate which respond to glutamate binding with fluorescence increase are still waiting to be developed.

## Gaba Biosensors

While glutamate is the primary excitatory neurotransmitter in the brain, GABA is the main inhibitory neurotransmitter. GABA interacts with the GPCR-C receptors. While the earlier GABA biosensors were “semisynthetic” (Masharina et al., 2012; Lecat-Guillet et al., 2017), recently, a fully genetically encoded GABA biosensor was developed (Marvin et al., 2019).

The first GABA semisynthetic biosensor, called GABA-Snift (Masharina et al., 2012), is based on the GABA_B_ receptor in which VFTD is N-terminally fused to the CLIP and SNAP tag peptide sequences. The SNAP and CLIP tags interact with the synthetic fluorophores forming a FRET pair. Moreover, in the GABA-Snift an antagonist of GABA, CGP71783, occupies a cleft between the lobes of the VFTD. When the cleft is occupied by CGP71783, FRET between synthetic fluorophores is weak; however, when GABA displaces CGP71783 the FRET enhances (Masharina et al., 2012). Another semisynthetic biosensor reports conformational changes in the heterodimeric GABA_B_ receptor that consist of GB1 and GB2 subunits. Similarly to GABA-Snift, in the GABA_B_ receptor-based biosensor the SNAP and ACP peptide tags are used. These tags are attached to VFTDs of the different GABA_B_ subunits. Fluorescein and Lumi4-Tb fluorophores bind the SNAP and ACP tags, respectively (Masharina et al., 2012; Lecat-Guillet et al., 2017).

The semisynthetic biosensors need adding of fluorophores, which complicates their use *in vivo*. Recently, a GABA-recognizing Pf622 protein from non-sequenced *Pseudomonas fluorescens* strain was used to engineer an intensiometric GABA biosensor, named iGABASnFR. In iGABASnFR, cpsfGFP is inserted into the Pf622 protein. As the membrane-anchoring sequences iGABASnFR utilizes an N-terminal immunoglobulin secretion signal and a C-terminal transmembrane domain of the platelet-derived growth factor receptor (PDGFR) (Marvin et al., 2019). Similarly to SF-iGluSnFR biosensor, iGABASnFR demonstrated good membrane localization, however, its response to neurotransmitter release was almost 10-fold smaller than of SF-iGluSnFR. In hippocampal acute slices, iGABASnFR detected GABA release caused by electric stimulation of *stratum radiatum* with sufficient signal-to-noise ratio. Yet, reliable recording of GABA release from individual synapses using iGABASnFR was challenging, whereas release of glutamate at individual synapses was easily detectable with SF-iGluSnFR.

## Acetylcholine Biosensors

Acetylcholine acts as fast point-to-point neurotransmitter in the peripheral nervous system and in neuromuscular junctions and as neuromodulator acting on the groups of neurons in the central nervous system. It is responsible for the adaptive behavior and coordinates responses of neuronal circuits in many brain areas (Picciotto et al., 2012). As a neuromodulator, acetylcholine influences neuronal excitability, synaptic transmission and synaptic plasticity. Moreover, acetylcholine coordinates firing of groups of neurons (Picciotto et al., 2012). It acts through the nicotinic receptors (nAchR), which are non-selective cationic channels, and muscarinic receptors (mAChR), which are GPCRs (Markovic et al., 2012) coupled to either Gq proteins (M1, M2, and M5 subtypes) activating phospholipase C or Gi/o proteins (M2 and M4 subtypes) inhibiting adenylate cyclase (Wess, 2003; Picciotto et al., 2012). Acetylcholine can act as inhibitory and excitatory neuromodulator, depending on the localization and type of muscarinic receptors. Action of acetylcholine on presynaptic mAChRs (M2/M4) is inhibitory whereas action on post-synaptic muscarinic receptors (M1/M5) is activatory (Picciotto et al., 2012). Action of acetylcholine on nicotinic ionotropic receptors in the brain is mostly neuromodulatory because nAChRs predominantly participate in coordination of neuronal firing (Picciotto et al., 2012).

M1-mAchR was used to develop the first genetically encoded acetylcholine biosensor. A FRET-based acetylcholine biosensor was engineered by inserting ECFP and EYFP into the third intracellular loop of the mouse M1-mAChR receptor. The resulting biosensor, called M1-cam5, retained the ability to stimulate downstream signaling of M1-mAchR (Markovic et al., 2012).

mAchRs were also used to develop intensiometric biosensors. For this, the longest third intracellular loop of mAchRs was replaced with the shorter third intracellular loop from the β_2_ adrenergic receptor, and cpEGFP was inserted in it, following random mutagenesis of the N- and C-termini of cpEGFP. After the first round of mutagenesis Jing et al. (2018) identified several best clones producing up to ∼70% fluorescence increase upon acetylcholine binding. The mutations from the found clones were rationally combined and the best biosensor variant was called GACh2.0 (Jing et al., 2018). In contrast to ratiometric M1-cam5 biosensor (Markovic et al., 2012) GACh2.0 exhibits weak coupling to downstream G-protein intracellular signaling, likely due to the replacement of mAchR third intracellular loop with the respective intracellular loop of the β_2_ adrenergic receptor.

Recently, the acetylcholine synthesis pathway was described in unicellular eukaryotes *Acanthamoeba* sp. (Baig et al., 2018). Earlier, a mAChR1 homolog was identified in *Acanthamoeba castellanii* (Baig and Ahmad, 2017). Likely, these unicellular eukaryotes can become a source of acetylcholine-binding domains for the development of novel acetylcholine biosensors.

## Dopamine Biosensors

Dopamine is primarily involved in the reward behavior, control of movement, emotion and cognition. Dysfunction of dopaminergic system is the cause of several mental disorders including Parkinsonism and autism spectrum disorder. Dopamine interacts with D1 and D2-like GPCR receptors. The important difference between D1 and D2-like receptors is their action on the production of the secondary messenger cAMP. Due to the coupling to the different types of G-proteins they either activate (D1) or inhibit (D2) cAMP production (Klein et al., 2019).

Existing methods of dopamine measurements are not well suited to detect changes of dopamine with both high spatial and high temporal precision during complex animal behavior. The widely used for dopamine measurements fast-scan cyclic voltammetry allows to measure longitudinal changes of dopamine in single recording locations, however, this technique is invasive and restricts animals (Rodeberg et al., 2017). Despite high temporal resolution of cyclic voltammetry, its spatial resolution is low and does not allow visualization of dopamine release from single neurons. Use of false fluorescent neurotransmitters (FFN) allows to visualize dopamine release with a single-neuron precision (Sames et al., 2013). However, it requires intracranial infusion of FFN, and is not applicable for longitudinal measurements (Dunn et al., 2018). In contrast, a genetically encoded biosensor for dopamine allows to measure dopamine transients in mice, zebrafish and flies with a high spatiotemporal precision for months (Patriarchi et al., 2018; Sun et al., 2018).

To engineer the intensiometric dopamine biosensor Sun et al. (2018). inserted cpEGFP in the third intracellular loop of several human dopamine receptors. Among them, a D1-cpEGFP chimera appeared to be the most promising. Changing the position of cpEGFP insertion and mutagenesis of linker residues led to the development of two biosensor variants, DA1m and DA1h. Both variants demonstrated rapid (∼60 ms for DA1m and ∼140 ms for DA1h) fluorescence increase in response to dopamine. A reversibility of the response was demonstrated by treatment with dopamine antagonist. Dopamine biosensors DA1m and DA1h (also called together GRAB_DA_) were orthogonal to the cell signaling and did not activate GPCR downstream pathways (Sun et al., 2018).

In another dopamine biosensor family, called dLight1, cpEGFP was inserted in the human D1 receptor (Patriarchi et al., 2018). The initial variant, which was obtained by inserting cpEGFP into the third loop of D1, showed a fluorescence decrease in response to dopamine. To engineer a positive-response biosensor Patriarchi et al. (2018) screened a library of the D1-based mutants in HEK293 cells to select a dLight1.1 variant, which exhibited the highest fluorescence increase in response to dopamine. An additional Phe129 mutation in the GPCR part of dLight1.1 resulted in a dLight1.2 variant with even higher dynamic range (Patriarchi et al., 2018).

Similar to GRAB_DA_ family, dLight1 biosensors do not interfere with G-protein signaling and, unlike natural D1 receptor, do not stimulate cAMP synthesis. Thus, the conversion of D1 to fluorescent biosensors blocked its ability to bind G-protein and trigger signaling (Patriarchi et al., 2018). Moreover, neither GRAB_DA_ nor dLight1 biosensors exhibit internalization, which is inherent to dopamine receptors (Sun et al., 2018).

## Sensing Norepinephrine Signaling

Norepinephrine, also known as noradrenalin, is a neurotransmitter that participates in the memory consolidation of emotionally arousing experiences (Cahill and Alkire, 2003). Norepinephrine is released by several brainstem nuclei including locus coeruleus (LC, a nucleus in the pons of brainstem) and is important for modulation of forebrain function. Release of norepinephrine by LC is associated with waking in both the cortex and hippocampus (Borodovitsyna et al., 2017).

Norepinephrine interacts with three types of adrenergic GPCR receptors, such as α1, α2 and β1. Receptors α1 and β1 activate phospholipase C and adenylyl cyclase whereas α2 mostly exerts inhibitory effect on cell signaling via suppression of adenylyl cyclase activity (Ramos and Arnsten, 2007).

To develop an intensiometric biosensor for norepinephrine Feng et al. (2019) inserted cpEGFP in the third intracellular loop of several adrenergic receptors including α2AR. Among all tested constructs, α2AR-cpEGFP preserved the membrane trafficking and, therefore, was selected for further optimization. The systematic truncation of the linker regions surrounding cpEGFP resulted in the family of norepinephrine biosensors (Table 1) consisting of GRAB_NE__1__m_ and GRAB_NE__1__h_ (Feng et al., 2019).

In another approach, a conformation-specific single-domain nanobody was proposed to probe activation of β2-adrenoceptor. The nanobody Nb80 recognizes β2AR only in its activated form, so that upon activation of β2AR the Nb80-EGFP fusion translocates from the cytoplasm to the plasma membrane. However, this type of translocation biosensors is difficult to implement *in vivo* (Irannejad et al., 2013).

All adrenergic receptors interact not only with norepinephrine but also with structurally similar epinephrine (Ramos and Arnsten, 2007). Thus, the development of norepinephrine-specific GPCR-based biosensor seems problematic if not impossible (Feng et al., 2018).

Bacteria are also able to sense norepinephrine. An example of bacterial receptor of norepinephrine is the histidine kinase QseC from enterohemorrhaghic *E. coli* strain. It was shown that a response of QseC to norepinephrine was blocked by norepinephrine antagonists (Clarke et al., 2006). Moreover, a homology modeling revealed the presence of typical for histidine kinases periplasmic signal-recognition domain, which is responsible for the norepinephrine binding (Clarke et al., 2006). Likely, use of this sensing domain as norepinephrine-binding template could result in the development of novel norepinephrine biosensors, similar to GltI-based biosensors for glutamate.

## Opioid Biosensor

Opioid receptors are GPCRs that are activated by endogenous opioid peptides and exogenous compounds. They play a key role in pain management, drug abuse and mood disorders. There are three major subtypes of opioid receptors, such as δ, μ, and κ (Shang and Filizola, 2015). Signaling of opioid GPCRs is not limited to the cell plasma membrane but to other cellular compartments (Irannejad et al., 2013) including endosomes and Golgi membranes (Eichel and von Zastrow, 2018). Studying opioid receptor signaling from different cellular locations was problematic because of the lack of relevant biosensors. To overcome this, Stoeber et al. (2018) developed a nanobody-based fluorescent biosensor. For that they selected a nanobody, which recognized only activated opioid receptors, and fused it to EGFP (Stoeber et al., 2018). This biosensor, called OR-sensor, allowed to detect difference between activation of opioid receptors by endogenous peptides and exogenous compounds, such as drugs. It was found that the peptide agonists produce a specific activation pattern initiated at the plasma membrane and propagated to endosomes after receptor internalization whereas drugs produce a different activation pattern by additionally causing opioid receptor activation in Golgi apparatus (Stoeber et al., 2018).

## Atp Biosensors

The function of the number of cellular metabolites depends on their location. An example is ATP, which is universal intracellular energy source and also a key purinergic signal that mediates cell-to-cell communication both in and between organs. Thus, targeting ATP biosensors to extracellular space allow to detect purinergic transmission (Burnstock, 2006).

This approach was implemented with an ATeam ratiometric biosensor for ATP. ATeam family of intracellular biosensors was the first developed by an Imamura group (Conley et al., 2017). These biosensors are composed of an ε subunit from a bacterial FoF1-ATP synthase that is inserted between ECFP and EYFP. ATP binding induces a conformational change that increases FRET between the ECFP donor and EYFP acceptor. Targeting ATeam3.10 using an immunoglobulin K leader sequence and a transmembrane anchor domain from the PDGFR to a surface of the plasma membrane turns it into an extracellular biosensor.

Similarly, an ε subunit of FOF1-ATPase from *Bacillus* PS3 was used to develop an intensiometric biosensor iATPSnFR. In this biosensor, cpEGFP is inserted between two α-helices of the ε subunit using two amino acid linkers from each side with expectation that conformational changes of the ε subunit might affect fluorescence. Both linkers were extensively mutated to maximize ATP-dependent fluorescence changes. To optimize expression of biosensor variants on the surface of HEK293 cells, EGFP was replaced with sfGFP and additionally mutated to reduce biosensor dimerization. The resulting iATPSnFR ATP biosensor exhibited efficient cell surface trafficking and 25% ΔF/F (Lobas et al., 2018).

## Biosensor for Glycine

Glycine acts as inhibitory neurotransmitter through ionotropic glycine receptors and as co-agonist of excitatory glutamate receptors of the NMDAR subtype. Recently, the first FRET-based glycine biosensor GlyFS was developed. For this, Zhang et al. (2018) used the Atu2422 protein from *Agrobacterium tumefaciens*. Atu2422 binds glycine, serine and GABA; however, rational design of the Atu2422 binding site allowed to significantly increase its specificity to glycine. A glycine-specific mutant of Atu2422, called AYW, was inserted between EGFP and mVenus FPs. The ratiometric response of this initial EGFP-AYW-mVenus construct to glycine was only 4%. To enhance it, Zhang et al. (2018) truncated the flexible linker between the AYW and EGFP and introduced a rigid (EAAAK)_4_ linker between AYW and mVenus. These modifications led to the increase of the dynamic range to 28%. Further elongation of the rigid linker caused the decrease of FRET efficiency. The resulting GlyFS biosensor was applied to detection of glycine transients in hippocampal acute slices. Since targeting of the GlyFS to the cell surface using immunoglobulin K leader sequence was ineffective, the biotin-streptavidin interaction was utilized. For that, acute brain slices were biotinylated, and the purified from expressing bacteria GlyFS biosensor with streptavidin was injected into the slices. Likely, use of the alternative membrane surface targeting peptides could result in the GlyFS delivery to the cell surface. Also, further engineering of glycine-binding AYW core could result in the development of an intensiometric glycine biosensor, as exemplified by use of GltI in both FRET and single-FP biosensors for glutamate (Table 1).

## Workflow for Engineering of Biosensors for Neurotransmitters

**FIGURE 3:**
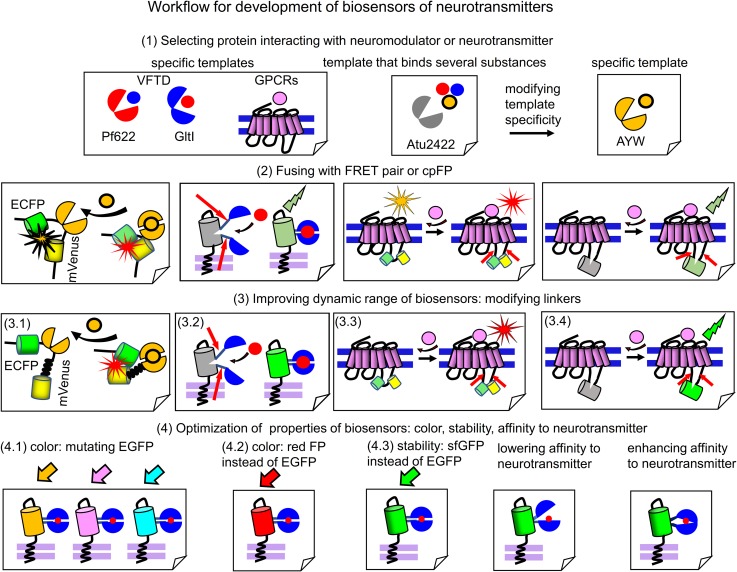
Workflow for engineering of biosensors. (1) Selection of a protein template, which interacts with the respective neurotransmitter or neuromodulator. From left to right: specific templates that bind relevant chemical substance with high specificity, such as GPCRs, GltI from *Escherichia coli* or Pf622 from *Pseudomonas fluorescens*. A promising template, such as Atu2422 from *Agrobacterium tumefaciens* that binds glycine, GABA and L-serine is also shown. Atu2422 mutant AYW binds only glycine and is used in glycine biosensor (Zhang et al., 2018). (2) Fusing of neurotransmitter-binding protein templates with FRET pairs of FPs or cpFPs. From left to right: Glycine-binding AYW is inserted between ECFP and mVenus; cpEGFP is inserted in the flexible region of GltI (Hires et al., 2008). FRET pair or cpFP is inserted in the third intracellular loop of GPCRs. Note the low dynamic range of relevant fusions. (3) Improving dynamic range of the initial fusions. (3.1) Deleting the flexible regions between ECFP and AYW in GlySF biosensor and inserting the rigid helical linker between AYW and mVenus (3.2) Mutagenesis of linkers joining VFTD domain and cpEGFP in a biosensor. (3.3 and 3.4) Mutagenesis of regions of the third intracellular loop of GPCRs in the point of insertion of FRET pair or cpFP. (4) Optimization of properties of resulting biosensors with high dynamic range. Manipulating color, stability and response dynamics. (4.1) Mutations, which change color of EGFP to yellow (mVenus), cyan (mTurquoise) and blue (mAzurite) are introduced in the cpEGFP-based biosensor to get a set of multicolor biosensors (Marvin et al., 2018). (4.2) EGFP is changed to mApple to get novel red fluorescent biosensor (Wu et al., 2018). (4.3) EGFP is changed to sfGFP to enhance biosensor stability. (4.3) Lowering affinity of the neurotransmitter-binding protein to the neurotransmitter allows to get biosensors able to detect fast neurotransmitter transients (Helassa et al., 2018; Marvin et al., 2018). (4.3) Enhancing affinity of neurotransmitter-binding protein allows to increase biosensor sensitivity to neurotransmitter (Marvin et al., 2018).

Modern biosensors for glutamate, GABA, acetylcholine, dopamine and glycine (Table 1) provide examples of the successful development of biosensors. Based on their engineering steps, we provide below the general workflow for development of biosensors for neurotransmitters and neuromodulators. The workflow consists of 4 steps outlined in Figure 3.

Step 1, an appropriate neurotransmitter or neuromodulator binding protein should be chosen. It can be VFTD or GPCR. As it was showed in engineering of biosensors for GABA (Marvin et al., 2019) and glycine (Zhang et al., 2018), a specificity of the selected sensing protein can become a problem. Natural Atu2422 protein binds both GABA and glycine, so that it is not possible to immediately use it for a biosensor specific to one of these neurotransmitters. Therefore, Zhang et al. (2018) mutated amino acid residues in the ligand-binding center of Atu2422 that resulted in a glycine-specific Atu2422 mutant, named AYW (Figure 3). Atu2422 sensing domain has been considered for GABA biosensor too, however, its low specificity prompted Marvin et al. (2019) to search for other GABA-binding proteins. That search resulted in the identification of Pf622 that interacts with GABA only (Marvin et al., 2019).

Step 2, the selected specific neurotransmitter or neuromodulator binding protein can be fused with either a cpFP or a FRET pair of monomeric FPs. For ratiometric FRET-based biosensors, this VFTD-containing sensing PBP is inserted between two FPs. For intensiometric biosensors, the cpFP is inserted in a flexible part of the VFTD-containing PBP, such as GltI in iGluSnFr (Marvin et al., 2013) or Pf622 in iGABASnFr (Marvin et al., 2019) biosensors. For both ratiometric and intensiometric GPCR-based biosensors, an insertion-point for FRET pair or cpFP is the same; it is the third intracellular loop that undergoes the most pronounced conformational changes upon GPCR activation.

Step 3, to efficiently report neurotransmitter changes several properties of the biosensor prototype obtained in the previous step should be improved. Fusing the neurotransmitter or neuromodulator binding protein with FRET pair or cpFP usually results in a biosensor variant with low dynamic range of FRET or fluorescence intensity changes upon binding the relevant substance. To improve dynamic range, a length and a composition of the linkers connecting FPs with the neurotransmitter-sensing domain (either VFTD or GPCR) should be modified by length and contents using either structure-based or random mutagenesis. For FRET-biosensors the linkers should provide an efficient separation of FPs in the non-bound state and optimally position FPs for high FRET in the bound state. For example, in GlySF glycine biosensor replacement of the flexible linker between AYW and FPs with the rigid helical linker (EAAAK)_4_ resulted in the sevenfold increase of dynamic range (Zhang et al., 2018). In other cases linkers were either subjected to random mutagenesis followed by screening, as in the dopamine biosensors (Feng et al., 2018; Patriarchi et al., 2018) or modified by stepwise deletions of amino acid residues, as in the acetylcholine biosensor (Jing et al., 2018).

Step 4, the resulting biosensors with high dynamic range can be subjected to several types of optimizations to improve parameters, such as color, stability, affinity and rate. Many intensiometric biosensors contain circular permutants of GFP, which can be changed to other FPs. For example, the cpsfGFP-based glutamate biosensor SF-iGluSnFR was converted into blue, cyan and yellow biosensors (Marvin et al., 2018) by introducing chromophore-modifying mutations in cpsfGFP (Figure 3). Changing cpEGFP in iGluSnFR to cpmApple resulted in the red glutamate biosensors R-iGluSnFR1 and R-ncpiGluSnFR1 (Wu et al., 2018). An ability of biosensor to monitor fast dynamics of neurotransmitter in synaptic cleft can be improved by manipulating of binding affinity to neurotransmitter in the sensing protein by mutating a ligand-binding site of the respective VFTD. For example, the decrease of affinity resulted in the development of fast glutamate biosensors, such as iGlu (Helassa et al., 2018) and SF-iGluSnFR/S72A (Marvin et al., 2018).

## Application of Neurotransmitter Biosensors *In Vivo*

Correlation of real-time neuronal activity with corresponding psychophysiological activities is one of the primary goals of neuroscience (Alivisatos et al., 2013). Since chemical transmission is the major communication pathway between neurons, precise detection of chemical transmission in neural circuits is required for achieving this goal. Chemical point-to-point transmission occurs extremely fast: depolarization of the post-synaptic neuron occurs within hundreds of milliseconds after glutamate release in the synaptic cleft. Chemical transmission underlies both unconscious and conscious behavior. Experimental objects perform complex psychophysiological tasks in 50–200 ms whereas perception of conscious experience requires 0.5–2 s (Korf and Gramsbergen, 2007).

Traditional molecular imaging methods, such as magnetic resonance imaging (MRI), proton MR spectroscopy (HMRS), and positron emission tomography (PET), has insufficient spatiotemporal resolution to efficiently visualize these fast events of chemical transmission. For example, PET has temporal resolution of few minutes and spatial resolution of few millimeters (Liang et al., 2015). Moreover, these techniques do not allow imaging of single neurons and separate neural circuits. However, spatially-precise longitudinal detection of fast neurotransmitter transients in the animal brain can be performed using modern optical techniques.

### Setups for *in vivo* Experiments

**FIGURE 4:**
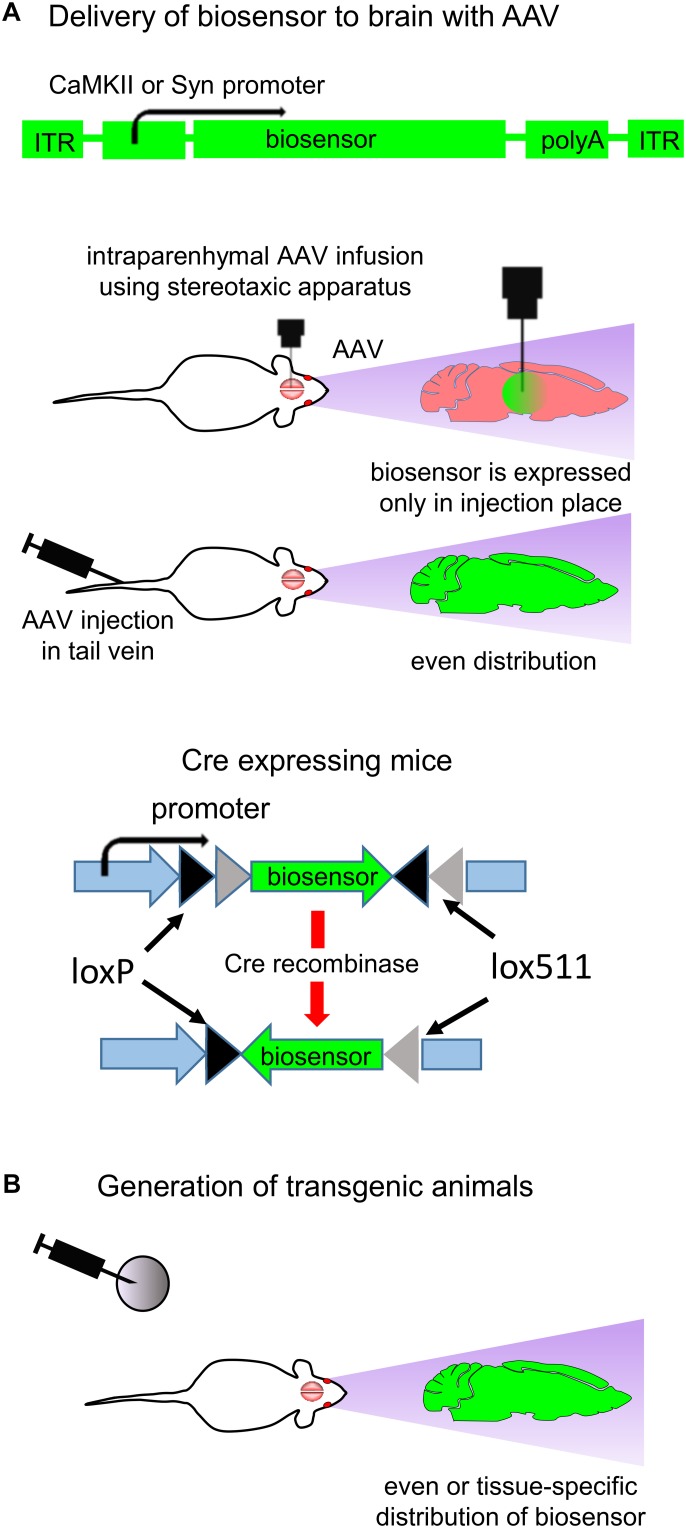
Expression of biosensors *in vivo*. **(A)** Delivery of biosensors to the animal brain using AAV particles. A typical structure of the AAV vector is shown (top). AAV transduction in adult animals can be performed by either stereotactic injection in the brain or tail injection (middle). Brain injection requires the stereotactic surgery, and the biosensor expression is restricted to the injection point. Via tail injection AAV are delivered to the whole body. The neuronal-specific promoters, such as for CaMKII kinase and Synapsin, provide biosensor expression in CNS (Stoica et al., 2013). Use of cell-specific Cre recombinase expression and AAV in which gene of biosensor is inverted and flanked with *loxP* and *lox511* sequences allows to achieve cell-specific biosensor expression (bottom) **(B)** Generation of transgenic animals allows to achieve either even or cell-specific expression of biosensor in the animal brain (Xie et al., 2016).

Design of *in vivo* experiment with fluorescent biosensors for neurotransmitters and neuromodulators, consists of a transduction of the relevant brain zone with adeno-associated virus (AAV) encoding a biosensor under specific promoters. AAV injection can be performed intravenously via either tail injection or using stereotactic injection, which requires surgery. Tail injection allows to achieve even AAV distribution in the rodent brain whereas stereotactic injection leads to expression of the neurotransmitter only at the injection place (Figure 4A; Boulaire et al., 2009; Stoica et al., 2013). In the case of tail injection a tissue-specific promoter is important to limit biosensor expression to certain tissue or subset of cells.

Common way to achieve neuron-specific biosensor expression is use of specific promoters, such as human *Synapsin1* (hSyn1) promoter or *CaMKII* promoter (Kugler et al., 2003). *Synapsin1* promoter ensures efficient neuronal targeting without expression in glial cells (Kugler et al., 2003). If expression of biosensor in glial cells is desirable then glial fibrillary acidic protein (GFAP) promoter can be used (Dashkoff et al., 2016).

Some promoters are able to limit transgene expression to one type of neurons only. For example, Hb9 promoter limits biosensor expression to motor neurons (Lukashchuk et al., 2016). mDlx enhancer placed before the minimal AAV promoter restricts transgene expression to GABAergic neurons (Dimidschstein et al., 2016; Wilson et al., 2017), and *Vglut2* promoter restricts transgene expression to glutamatergic neurons (Borgius et al., 2010). For detailed description of the relevant neuronal-specific promoters we refer readers to several reviews (Shevtsova et al., 2005; Hioki et al., 2007; Delzor et al., 2012; Dashkoff et al., 2016).

Generation of a transgenic animal line is another way to achieve cell-specific biosensor expression in the mammalian brain (Figure 4B; McGirr et al., 2017). Biosensor expression is defined to the specific subset of neurons if mice with cell-specific expression of Cre recombinase are used. In this case, mice are injected with AAV in which the biosensor encoding sequence is inverted and flanked with *loxP* and *lox511* sequences (Figure 4A; bottom). Cre recombinase recognizes the *lox* sequences, excises and inverts the biosensor gene. For example, expressing Cre recombinase under the glutamatergic neurons specific *Vglut2* promoter allows to confine the gene expression to the excitatory glutamatergic neurons (Borgius et al., 2010).

**FIGURE 5:**
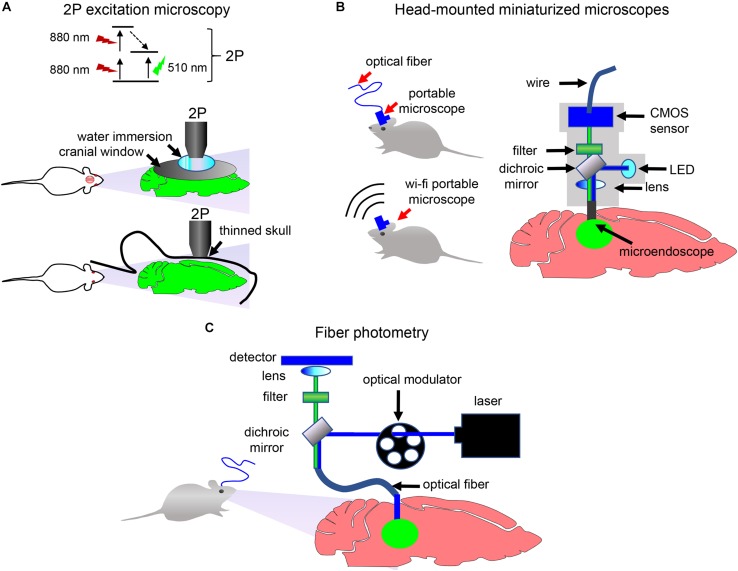
Detection and imaging of neuronal activity *in vivo*. **(A)** 2P excitation microscopy of immobilized animals. Principle of the 2P excitation is depicted (top). Non-invasive 2P imaging of the brain in immobilized animals through the cranial window (middle) and through the thinned skull (bottom) is shown. **(B)** Schematics of the portable single-photon microscope for imaging of neurotransmission in freely moving animals. Imaging data can be transferred to the detection device using either optical fiber or wirelessly. **(C)** Schematics of the fiber photometry setup for detection in freely moving animals. Optical fiber is implanted in the animal brain. Optical modulator determines excitation light frequency. Fluorescent signal from biosensor is collected with the same frequency using photodetector and normalized to a signal at times when excitation light is turned off (Resendez and Stuber, 2015).

Delivery of biosensor can be followed by the visualization of transmitter-specific events in the brain of behaving animals with 2P microscopy, miniaturized head-mounted microscopes or fiber photometry (Figure 5; Helmchen et al., 2001; Guo et al., 2015; Brunert et al., 2016; Ozbay et al., 2018).

When choosing how image acquisition should be performed, two important issues should be considered. If study is performed in immobilized animals, then imaging can be done using a stationary 2P microscope. The 2P microscopy can be performed via two types of imaging windows. In one type, a thin-skull window technique is used in which the skull is thinned down to a thickness of ∼15 μm. In another type, a part of skull is removed and a glass cranial window is places instead (Figure 5A). Both window techniques have advantages and disadvantages. The thinned-skull window is less invasive and allows immediate chronic imaging after surgery and long imaging intervals. Open-skull window allows imaging of deep brain layers (Yang et al., 2010). Thinned-skull and open-skull window techniques are compared in details in several reviews (Yang et al., 2010; Isshiki and Okabe, 2014).

If detection of neurotransmitter transients in freely behaving animals is desirable, then a miniaturized head-mounted microscope or a fiber photometry can be used (Figures 5B,C). A number of miniaturized devices has been developed by different research groups (Aharoni et al., 2019). While usually a miniaturized microscope is connected with the detection device using an optical fiber, wireless microscopes became recently available too (Liberti et al., 2017). In contrast to single-photon head-mounted microscopes that frequently require invasive brain surgery for inserting optical objectives or prisms in the brain, 2P miniaturized microscopes allow non-invasive deep-brain imaging via thinned skull or, with limited skull surgery, via cranial window (Silva, 2017). Moreover, modern 2P head-mounted microscopes allow high-resolution imaging of cortex with visualization of individual dendrites and dendritic spines (Silva, 2017; Ozbay et al., 2018).

Similarly to miniaturized microscopes, a fiber photometry (Figure 5C) enables detection of fluorescence in the brain of freely moving animals. However, it requires implantation of an optical fiber in the animal brain and, opposed to the above microscopy approaches, lacks single-cell resolution. Nevertheless, simplicity of fiber photometry instrumentation and high sensitivity of detection of neuronal activities makes it attractive to researchers (Resendez and Stuber, 2015). Multicolor fiber photometry is also available, allowing readout of several biosensors simultaneously. Moreover, a wireless fiber photometry was recently developed to detect biosensor responses in the brain of non-tethered animals (Lu et al., 2018).

### Examples of Applications of Biosensors for Neurotransmitters

Biosensors for neurotransmitters are used in animals in a wide range of studies, from simple responses to sensory stimuli to complex animal behaviors in models of mood disorders.

#### Mapping Neurotransmitter Transients in Cortex of Behaving Animals

**FIGURE 6:**
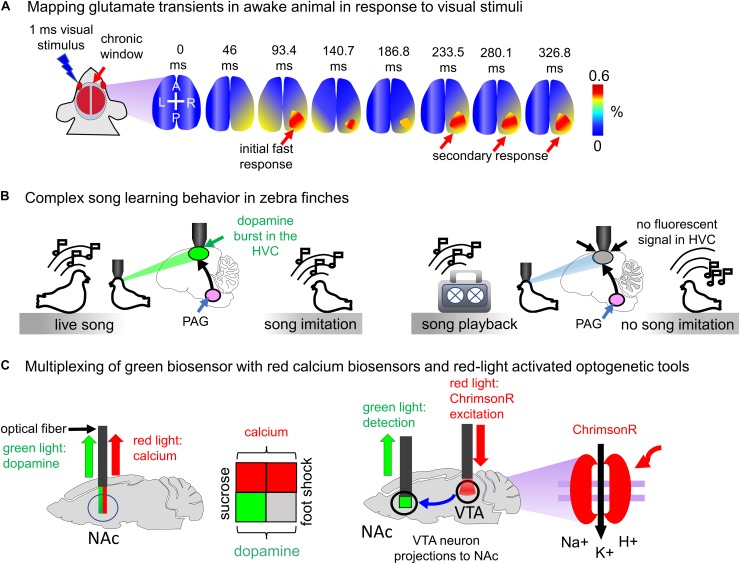
Visualization of neurotransmitter transients *in vivo*. **(A)** Mapping glutamate transients in the mouse brain is performed through the chronic cranial window. One millisecond light visual stimulation induces an initial fast response followed by a clearly separated secondary response in awake Emx-CaMKII-iGluSnFR mouse (Xie et al., 2016). **(B)** Complex song learning in zebra finches is outlined. Left: juvenile zebra finch is exposed to the living adult male tutor. The song causes dopamine bursts in the high vocal center (HVC) of the juvenile bird, resulting in song learning. Right: juvenile zebra finch is exposed to the playback of the song. Dopamine bursts in the HVC of juvenile bird are not detected, and consequently, song learning does not occur (Tanaka et al., 2018). **(C)** Spectral multiplexing of green fluorescent dLight1.1 with either red fluorescent jRGECO1a biosensor or channelrhodopsin ChrimsonR activatable with red light. Left: both dopamine and calcium signals are detected in the mouse brain using multicolor fiber photometry. The sucrose application causes both dopamine transients and neuronal activity in the NAc (green and red squares), whereas the foot shock increases calcium but not dopamine transients (gray and red squares). Right: ChrimsonR channelrhodopsin is selectively expressed in the VTA of the mouse brain. Activation of ChrimsonR with red light causes the dopamine release in the NAc. The VTA neuron projections to NAc are shown with the arrow.

Neurotransmitter biosensors allow precise spatiotemporal mapping of neurotransmission in the brain of animals. For example, Xie et al. (2016) used iGluSnFR to determine high-frequency mesoscale intracortical maps. In this study iGluSnFR enabled to resolve temporal features of sensory processing in both anesthetized and awake mice. The fast glutamate transmission events on 13–200 ms timescale in response to sensual stimuli, such as touching whiskers, skin on the fore- and hind limbs, and visual stimuli, were imaged (Figure 6A; Xie et al., 2016).

iGluSnFR was also used to localize task-specific glutamate events in the primary motor cortex of mice. iGluSnFR was delivered to the mouse motor cortex using AAV particles, and the brain of mice was imaged in rest and upon running (while head of running mice was fixed). It was found that in the brain of resting mice the repetitive glutamate transients are observed in dendritic spines. Running increased frequency of these events twice over 8 s of running. Extremely high spatiotemporal resolution in this study demonstrates the utility of iGluSnFR for the precise mapping of glutamate release events (Marvin et al., 2013).

#### Complex Song Learning in Zebra Finches

Juvenile zebra finches copy songs of the living bird adult tutors only if they interact with them and fail to reproduce songs played to them through a speaker (Figure 6B). To elucidate how juvenile birds detect a difference between the tutor and speaker Tanaka et al. (2018) expressed intensiometric dopamine biosensor GRAB_DA__1__*h*_ in the neurons of high vocal center (HVC) and imaged dopamine transients using 2P microscopy. He has found that only interaction with the song of live tutor caused dopamine secretion by the neurons of the periaqueductal gray (PAG). Even playback of the song from the recent tutor failed to evoke similar activity. Thus, the single-FP neurotransmitter biosensor allowed to visualize dopamine transients with the single-neuron precision and to establish connection between dopamine secretion by the PAG neurons and transmission of vocal behaviors from one bird to another (Tanaka et al., 2018).

In the same study the PAG neurons were excited optogenetically using ChR2 channelrhodopsin actuator expressed in the HVC. Interestingly, the excitation of the HVC neurons via ChR2 in combination with playback of the song from the speaker resulted in the successful learning by juvenile birds (Tanaka et al., 2018). Dopamine blockers reduced the effect. However, optogenetic excitation was not combined with the simultaneous analysis of dopamine transients because ChR2 is excited by the same light used for visualization of EGFP-based biosensors.

#### Multiplexing of dLight1.1 With Red-Light Excited Probes

The availability of red fluorescent calcium biosensors and red-shifted channelrhodopsins provides possibility either to simultaneously detect neurotransmitter transients and neuron activity or to combine optogenetic excitation of certain neurons with detection of neurotransmitters released by their terminals. Patriarchi et al. (2018) combined visualization of dopamine transients with calcium imaging using jRGECO1a red biosensor. It has been shown that mice consuming water containing sucrose (reward) show both dopamine and calcium peaks in the nucleus accumbens (NAc) region. However, mice subjected to foot shock demonstrated only calcium spikes in the NAc (Figure 6C; Patriarchi et al., 2018).

In the same study dLight1.1 was combined with the red-light excited channelrhodopsin ChrimsonR (Figure 6C). Dopaminergic neurons in the ventral tegmental area (VTA) send projections to the NAc. dLight1.1 was delivered in the mouse brain using AAV particles, and ChrimsonR was selectively expressed in the VTA. Photostimulation of neurons in the VTA enabled detection of individual peaks of dopamine transients in the NAc region.

#### Imaging Neurotransmission in Animal Behavioral Models

Dysregulation of neurotransmission underlies the number of brain diseases, making the biosensors useful in various animal models of human neuropsychiatric disorders. The animal models of human CNS disorders proved their effectiveness in studies of Parkinsonism (Blesa and Przedborski, 2014), major depressive disorder (Morozova et al., 2016; Zorkina et al., 2019), autism (Chesselet, 2005) and many others reviewed elsewhere (Keifer and Summers, 2016).

For example, the glutamate intensiometric biosensor iGluSnFR was used to study antidepressant activities of ketamine (McGirr et al., 2017). Transgenic mice expressing iGluSnFR (Figure 4B) were subjected to social defeat model of depression and then treated with ketamine. Glutamate transients were imaged non-invasively using the thin-skull window technique. Longitudinal tracking of iGluSnFR signal revealed that social defeat caused the network-wide glutamate functional hyperconnectivity in animals whereas injection of ketamine reduces this effect.

In another study, the GRAB_DA_ biosensor for dopamine was applied to detect endogenous dopamine release during Pavlovian conditioning in immobilized and freely moving mice (Feng et al., 2018). The water-restricted mice were trained to associate a brief auditory cue with reward (a drop of water). Monitoring fluorescence changes of GRAB_DA_ in these trained animals allowed to visualize dopamine release in response to the reward-predictive cue (sound). Also, the dopamine biosensor was used to study dopamine dynamics during naturally rewarding social behaviors, such as courtship and mating. It was confirmed that introduction of the sexually receptive mouse female into the home cage of the male promoted dopamine release during mating.

Moreover, given the important role of dopaminergic transmission in reward and pleasure behavior (Bressan and Crippa, 2005) it will be advantageous to apply dopamine biosensors in animal models of depression, similarly to iGluSnFR.

## Conclusion

Recently developed fluorescent biosensors for glutamate, dopamine, acetylcholine, adrenaline and GABA allow to detect neuronal activity *in vivo* with high spatiotemporal precision. Single-FP-based intensiometric biosensors represent the most useful group of the biosensors because they are monochromic and have the higher dynamic range than FRET-based, thus, enabling spectral multiplexing with other biosensors or optogenetic tools and imaging of neuronal activity *in vivo*, respectively.

In spite of many advantages, there are two major limitations of the biosensors for neurotransmitters and neuromodulators. One limitation is that these biosensors may influence dynamics of neurotransmitters and neuromodulators in the brain by binding them. The similar problem and possible ways of its resolution were recently described for calcium biosensors (McMahon and Jackson, 2018). The other limitation is related to use of the biosensors in the human brain. Although nervous system is considered immunologically tolerant, but expression of proteins from bacteria and invertebrates may cause immunological response. Currently, expression of bacterial channelrhodopsins is restricted to human eyes in vision restoration (Baker and Flannery, 2018). However, heterologous expression of bacterial proteins may become a subject of immunogenicity (Maimon et al., 2018).

We foresee the following future directions in the development and applications of biosensors for chemical transmission.

First, the major characteristics of the existing fluorescent biosensors, such as selectivity, stability, sensitivity, kinetics, reversibility and dynamic range (Shang and Filizola, 2015), will be enhanced and optimized for specific applications in the mammalian brain.

Second, the availability of the neurotransmitter- and neuromodulator-binding proteins in unicellular and multicellular organisms, including diverse bacterial ABC-transporters (Moussatova et al., 2008), GABA-binding malate transporters in plants (Ramesh et al., 2015), and monoamine-binding transporters in worms (Ribeiro and Patocka, 2013), allows to anticipate that they will be used as molecular templates to engineer novel biosensors. Likely, search of these templates can be alleviated using machine learning approaches, such as deep learning. Machine learning is based on computer algorithms, which are able to learn automatically to distinguish between various datasets, for example, between two sets of images (Majaj and Pelli, 2018). Deep machine learning is based on complex multi-layered artificial neural networks (Miotto et al., 2018; Mehta et al., 2019). Deep learning approaches are already used to predict interaction of a chemical substance, like drug, with a target protein. Likely, deep learning approaches for prediction of drug-target interactions (Anusuya et al., 2018; Lee et al., 2019) and to annotate protein functions (Sureyya Rifaioglu et al., 2019) can be adapted to predict interaction of neurotransmitters or neuromodulators with proteins with unknown function. Thus, novel specific neurotransmitter-binding proteins can be found while limitations derived from use of GPCRs or finite number of bacterial proteins used in biosensor engineering could be overcome. Use of deep learning for identification of such proteins *in silico* will reduce laborious and time-consuming search for neurotransmitter-binding proteins *in vitro*.

Third, the available biosensors have fluorescence readout mainly in the green range of light spectrum, with few exceptions in the red range. We anticipate that more red and, moreover, far-red and near-infrared fluorescent biosensors for neurotransmitters will be developed based on the modern red (Shcherbakova et al., 2015), far-red and near-infrared FPs (Chernov et al., 2017; Oliinyk et al., 2017; Shcherbakova et al., 2018). Far-red and near-infrared light is less cytotoxic, penetrates animal tissues deeper, and exhibits less scattering. Moreover, far-red and near-infrared biosensors will allow cross-talk free simultaneous use of common optogenetic tools and major calcium biosensors.

Fourth, we hypothesize that processing of the data obtained in imaging experiments will benefit from deep learning methods. Neurotransmitter and neuromodulator biosensors allow *in vivo* detection of large neural populations during weeks with single-neuron and single-spike resolution, similar to calcium biosensors (Pnevmatikakis, 2019). Several deep learning-based techniques for calcium imaging (Stringer and Pachitariu, 2019) can be adopted for processing of data obtained in experiments with neurotransmitter imaging. For example, an artificial neural network STNeuroNet was recently used to identify and segment active neurons expressing calcium biosensor (Soltanian-Zadeh et al., 2019). Likely, the similar technologies will be applied for analysis of large datasets of neurotransmitter and neuromodulator imaging.

Fifth and last, much wider implementation of biosensors for neurotransmitters and neuromodulators for mapping of brain activity can be anticipated. Correlation between brain activities and behavior makes possible prediction of motor or cognitive functions out of imaging data (Li et al., 2019). Data obtained by imaging of fluorescent biosensors for neurotransmitters and neuromodulators in animal brain can also be used to predict motor and cognitive function in animal models. For example, calcium imaging data recorded by 2P microscopy in rodent brain were used to predict features of upcoming movement (forelimb reach) in mice. For that, the authors collected calcium imaging signal from motor cortex while mice were performing a two-dimensional lever reaching task. Obtained calcium imaging data were used to train deep learning model to predict forelimb movement direction in mice. This deep learning model was then used to determine the motion direction based on imaging of calcium in the motor cortex (Li et al., 2019). We hypothesize that this approach can be expanded to the prediction of motion direction out of neurotransmitter and neuromodulator biosensor imaging. Such data can be further applied in the field of brain-computer interface (Andersen et al., 2014; Li et al., 2019).

## Author Contributions

All authors contributed to the writing and reviewing of the manuscript.

## Conflict of Interest

The authors declare that the research was conducted in the absence of any commercial or financial relationships that could be construed as a potential conflict of interest.
